# Risk of infectious mononucleosis is not associated with prior infection morbidity

**DOI:** 10.3389/fepid.2025.1518559

**Published:** 2025-03-12

**Authors:** Klaus Rostgaard, Ragnar Kristjánsson, Olafur Davidsson, Jojo Biel-Nielsen Dietz, Signe Holst Søegaard, Lone Graff Stensballe, Henrik Hjalgrim

**Affiliations:** ^1^ Danish Cancer Institute, Danish Cancer Society, Copenhagen, Denmark; ^2^ Department of Epidemiology Research, Statens Serum Institut, Copenhagen, Denmark; ^3^ Department of Congenital Disorders, Statens Serum Institut, Copenhagen, Denmark; ^4^ Department of Pediatrics and Adolescent Medicine, Rigshospitalet, Copenhagen University Hospital, Copenhagen, Denmark; ^5^ Department of Hematology, Copenhagen University Hospital, Copenhagen, Denmark; ^6^ Department of Clinical Medicine, Copenhagen University, Copenhagen, Denmark

**Keywords:** Epstein–Barr Virus, immune system, infections, infectious mononucleosis, puberty, Bayes factor

## Abstract

**Background:**

The probability of presenting with infectious mononucleosis (IM) upon primary Epstein–Barr virus infection increases dramatically at the start of puberty. Aiming to understand why that is, we assessed whether the number of infection-related health events during two specific time periods−ages 10–12 years (pre-teen window) and the three most recent years (recent window)−could predict the likelihood of individuals aged 13–19 years developing IM.

**Methods:**

We used sibship-stratified Cox regression to mitigate socio-demographic confounding and bias. Consequently, we only followed members of IM-affected sibships aged 13–19 years between 1999 and 2021 for IM, based on information from complete nationwide Danish administrative and health registers. Estimates were further adjusted for sex, age, birth order (1, 2, 3+) and sibship constellation [number of siblings and their signed (older/younger) age difference to the index person]. Infection-related health events defining the exposures considered were either a category of antimicrobial prescription, or a hospital contact with an infectious disease diagnosis. We measured evidence/probability of the associations using asymptotic Bayes factors, rather than using *p*-value based testing.

**Results:**

The adjusted hazard ratio (HR) for IM with 95% confidence limits for an additional antimicrobial prescription in the pre-teen exposure window was [1.01; 0.98–1.04], and the corresponding adjusted HR for an additional antimicrobial prescription in the recent exposure window was [1.02; 0.99–1.06].

**Conclusions:**

IM was not preceded by unusual numbers of infections. Small effect sizes, together with small variation in exposure, did not render the assessed exposures useful for predicting IM for public health or the clinic.

## Introduction

Most individuals become infected with Epstein–Barr Virus (EBV), which then establishes a persistent and mostly latent infection in the host (1). Primary EBV infection usually occurs either in early childhood (ages 0–2 years) or during teenage years (1). Primary EBV infection occurring in teenage years or later often presents as infectious mononucleosis (IM), which is comparatively rare at younger ages (1). Recent investigations have shown that the probability of presenting with IM upon primary EBV infection (the attack rate) increases dramatically during puberty (1). As IM is caused by an overreaction of the immune system, this change in attack rate is most likely rooted in the changing capabilities and reactivity of the immune system, specifically an exaggerated CD8+ response (1, 2). Furthermore, puberty brings a maturation of the immune system, at least with respect to expression and production of immunoglobulin G antibodies, co-stimulatory molecules and cytokine production by type 2 dendritic cells and plasmacytoid dendritic cells, as well as boosting T helper 1 (Th1), Th17, and T follicular helper immunity (3).

Firstly, understanding these rapid and age-related changes in the IM attack rate may be helpful in combating IM and, secondly, may yield clues to understanding similar changes in the occurrence of other EBV-related diseases such as multiple sclerosis (4), Hodgkin lymphoma (5), and inflammatory bowel disease (6, 7) in corresponding age groups. Such an understanding would strengthen the foundation for designing EBV vaccines targeting long-term consequences of primary EBV infection; i.e., in situations where experimental verification in randomized clinical trials become infeasible, and the application of such vaccines has to rely on a consensus in the broad scientific community that all relevant aetiological details were known, that the vaccine would work as intended, and at least not harm in unforeseen ways (8, 9).

Cohort studies of teenagers and young adults with frequent measurements of immune-related biomarkers before and shortly after EBV infection and IM are practically non-existent (9–12). This is the case, even though relatively small studies would be large enough to yield statistically robust inference regarding the kinetics of primary EBV infection and what drives the presentation to be that of IM, given how common IM is (1). Absent such data, we set out to assess whether counts of specific types of immune-related health events, e.g., antimicrobial prescriptions filled, in exposure windows before and during adolescence could be predictive of IM in teenage years. Similar methodology has been used with some success in aetiological research regarding diseases in children and adolescents, and is not always based on *a priori* hypotheses (13–16). Thus, eventual associations between counts of immune-related health events and IM risk may provide more preliminary evidence for a link between puberty-associated biological processes and changes in the IM attack rate (1, 3).

## Methods

### Cohort and design

All data used in this study were obtained from Danish nationwide and complete administrative and health registers (17, 18). We followed individuals for IM from age 13 to 19 years while resident in Denmark and before 1st January 2022. We assessed counts of immune-related health events as exposures in two three-year wide exposure windows. The pre-teen exposure window spanned ages 10–12 years, while the recent exposure window ended three months before their attained age. This 3-month exclusion period was designed to sidestep any issues of reverse causation, as the incubation period from EBV infection to fulminant IM is about 6 weeks (1). Exposures and outcomes originated from the Danish National Patient Register, covering the period since 1977 (19), and from the Danish National Prescription Register, established in 1994, recording prescriptions for children under their own identity (rather than their legal guardian's) since 1996 (20, 21). Information on family relations, residence, sex, birth dates, and civil status, allowing for the calculation of all needed derivatives thereof, e.g., age, birth order, and sibship constellation, was obtained from the Danish Civil Registration System, which also through the unique personal identification numbers underpins identity-secure linkage between registers (17, 18).

To mitigate socio-demographic biases, we matched on sibship and therefore only needed to follow individuals from IM-affected sibships (9). We only followed individuals with known exposures, i.e., residents of Denmark throughout the exposure window, requiring the latter to start after December 31st, 1995. Thus, in order to have complete information in the data available to us, follow-up was restricted to calendar years from 1999 to 2021.

### Outcome

We identified IM cases as individuals having a main, secondary, or underlying diagnosis code of B27 [international classification of diseases, 10th revision (ICD-10)] or 027 (ICD-8) in a hospital contact in the Danish National Patient Register (19), taking the earliest admission date for such a contact to be the date of IM diagnosis.

### Exposures

For all participants, we obtained information on antimicrobial prescriptions [anatomical therapeutic chemical classification system (ATC) codes J01A, J01C-J01G, J01M, J01X, J02A, J04A, J05A, P01A, P02C] from the Danish Prescription Register, covering the period since 1996 (20, 21). The prescriptions included antibacterials, antimycobacterials, antifungals, antivirals, antiprotozoals, and antihelmintics (16). The vast majority of these products were intended for treating respiratory infections (ATC codes: J01CA04, J01CE02, J01CR02, J01FA) (22). We likewise obtained information on hospital contacts involving main, secondary or underlying diagnoses for infectious diseases (ICD-10 chapters A and B) from the Danish National Patient Register (19). Both inpatient contacts, available from 1977 onwards, and outpatient contacts, from 1994 onwards, were considered.

We tallied observed counts of each assessed health event per exposure window. Inspired by previous work (9), we considered health events as per three definitions: (a) any infection diagnosis from a hospital (excluding IM), (b) any antimicrobial prescription mentioned above, and (c) the specialization to any antiviral prescription (ATC code J05A).

### Statistical methods

To mitigate socio-demographic biases we matched on sibship, i.e., used stratified Cox regression with calendar time as the underlying time scale and sibships, defined by a common mother and father, as strata. Given the design of the study, compared siblings could be at most 7 years apart in age. Crude analyses in this model were accompanied by analyses further adjusted for the important predictors of IM: age, sex, sibship constellation [number of siblings and signed (older/younger) difference in age to the index person], and birth order to avoid bias towards the null in the Cox regression model (23, 24). Part of this adjustment was performed by entering an offset, to avoid technical estimation problems (12). We calculated sex- and age-specific empirical IM incidence rates (events/person years at risk) during follow-up in sibships with IM, with age in 1-year categories and entering the log of the relevant rates as part of this offset. The other part of this offset was the siboffset (12), modelling the effect of a given time-dependent sibship constellation as siboffset = Σ*_k_* log (HR*_k_*) × pred*_k_* using the predictors and hazard ratios from (12). This modelling in turn was based on number of siblings of a certain age (0,1,2,3 years) and number of siblings with a certain age differential to the index child in eight categories, and an interaction between the age of each sibling and the age of the index person. Thus, adjustment was performed by entering this time-varying offset and further adjusting for birth order in categories of 1, 2, and 3 or more.

All analyses were performed using the SAS statistical software package (version 9.4 SAS Institute, Cary, NC, USA) and the stratify macro (25). Ninety-five percent confidence intervals (CIs) were based on Wald tests. The evidence for association between exposure and outcome was assessed by asymptotic Bayes factors (BFs), providing an objective tool for directly measuring (relative) evidence for the alternative and the null hypotheses, i.e., BF = (evidence for H1 of an association)/(evidence for H0 of no association) (26). Asymptotic Bayes factors and related functions are available in the EpiForsk R package (27), but here we just calculated BF in SAS, since all other data processing and analysis was performed in SAS.

## Results

The association between number of infection-related health events and subsequent IM risk was estimated among 10,840 children of age 13–19 years, comprising 5,123 IM affected sibships in Denmark who experienced 5,228 IM events in the period 1999–2021 based on complete, nation-wide registers.

Considering the two-by-three *a priori* defined exposures (models 10–12, 20–22) we only found association to be likely between recent antimicrobial use and IM (Table 1; panels A and B). As a first attempt to pinpoint the basis of this association we then considered two-by-four further exposures (models 13–16, 23–26), covering the four most common types of antibacterials: tetracyclines (ATC code J01A), penicillins (ATC code J01C), Sulfonamides (ATC code J01E) and macrolides (ATC code J01F). This yielded similar effect estimates (Table 1; panels A and B), prompting us to search for a bias or subpopulations that could be responsible for the positive association between recent antimicrobial use and IM risk, specifically by stratifying our results by sex and calendar period of the outcome (Table 1; panel C). This effort pinpointed the last half of the study period among girls as seemingly the only stratum showing an association. We then assessed the distribution of antimicrobials use by IM case status (Table 2). There was no suggestion of heterogeneity in antimicrobial exposure according to later IM-status among girls in the period before 2010 (BF = 0.62), but clearly more use of antimicrobials among some of those who ended up as IM cases in girls in the period from 2010 and onwards (BF = 8.95).

**Table 1:** Hazard ratios (HRs) with 95% confidence limits and associated Bayes factors (BFs) for IM per additional exposure event.

Exposure	Total exposure events	HR (95% CI) crude	HR (95% CI) adjusted	BF crude	BF adjusted
A: Pre-teen exposure					
10 Infection hospital contacts	163	1.01 (0.79–1.30)	1.00 (0.77–1.30)	0.45	0.45
11 Antimicrobials	7,902	1.02 (0.99–1.05)	1.01 (0.98–1.05)	0.83	0.61
12 Antivirals	175	1.07 (0.91–1.27)	1.10 (0.92–1.31)	0.60	0.67
13 Tetracyclines	50	1.12 (0.79–1.59)	1.04 (0.73–1.49)	0.53	0.46
14 Penicillins	5,620	1.03 (0.99–1.07)	1.02 (0.98–1.07)	0.90	0.73
15 Sulfamides	205	1.12 (0.92–1.36)	1.09 (0.89–1.33)	0.76	0.59
16 Macrolides	871	1.01 (0.92–1.12)	1.00 (0.90–1.11)	0.47	0.45
B: Recent exposure					
20 Infection hospital contacts	239	0.98 (0.80–1.21)	0.97 (0.78–1.22)	0.46	0.46
21 Antimicrobials	12,868	1.03 (1.01–1.05)	1.03 (1.01–1.05)	11.49	5.58
22 Antivirals	368	1.00 (0.91–1.11)	1.01 (0.92–1.12)	0.45	0.46
23 Tetracyclines	1,270	1.03 (0.97–1.09)	1.03 (0.96–1.09)	0.65	0.59
24 Penicillins	7,751	1.06 (1.02–1.09)	1.05 (1.01–1.09)	47.28	7.27
25 Sulfamides	600	0.93 (0.83–1.05)	0.94 (0.83–1.07)	0.76	0.64
26 Macrolides	1,827	1.03 (0.97–1.09)	1.03 (0.97–1.10)	0.68	0.70
C: Recent exposure					
30 Antimicrobials (females)	8,049	1.04 (1.02–1.06)	1.02 (1.00–1.05)	38.03	1.37
31 Antimicrobials (males)	4,819	1.00 (0.96–1.03)	1.03 (0.99–1.06)	0.47	1.04
32 Antimicrobials (<2010)	5,378	1.00 (0.97–1.03)	1.00 (0.96–1.03)	0.46	0.46
33 Antimicrobials (2010+)	7,490	1.06 (1.03–1.09)	1.05 (1.02–1.08)	228.47	58.07
34 Antimicrobials (females and 2010+)	4,768	1.06 (1.03–1.09)	1.04 (1.01–1.07)	327.31	5.94
35 Antimicrobials (males or <2010)	8,100	1.00 (0.97–1.02)	1.01 (0.98–1.04)	0.45	0.59

**Table 2:** Recent antimicrobial use in females, by case status (IM+) and time period.

<2010						2010+				
Exposure	IM+ *n*	IM+ %	IM− *n*	IM− %	OR 95% CI	IM+ *n*	IM+ %	IM− *n*	IM− %	OR 95% CI
0	503	44.0	423	40.6	1.00 (Ref)	697	41.6	744	46.9	1.00 (Ref)
1	261	22.8	253	24.3	0.87 (0.70–1.08)	419	25.0	356	22.4	1.26 (1.05–1.50)
2	156	13.6	130	12.5	1.01 (0.77–1.32)	223	13.3	216	13.6	1.10 (0.89–1.36)
3+	224	19.6	235	22.6	0.80 (0.64–1.00)	338	20.2	272	17.1	1.33 (1.10–1.60)

## Discussion

### Main findings and study rationale

Most observed effect sizes were very small, and those remaining were restricted to the stratum of recent exposures in girls in the second half of the study period (Table 1), making it unlikely to represent a true biological phenomenon. These small effect sizes, combined with the small variation in exposures (Table 1 and Table 2), rendered the considered exposures irrelevant for prediction or public health *per se*.

Being without access to relevant biomarker measurements (9–12), we attempted to answer immunological questions using only plain epidemiology. Admittedly, this is courageous; but it is not impossible. On earlier occasions, we have, for example, described biologically meaningful excess use of antimicrobials (and variation in use between different types of antimicrobials) up to several years prior to diagnosis of chronic lymphocytic leukemia, as well as increased use of antimicrobials in children and grandchildren of chronic lymphocytic leukemia cases (14). We have also found the incidence of subtypes of Hodgkin lymphoma at age 10–25 years to be associated with antimicrobial use at age 0–9 years in biologically meaningful ways (13). Further, with varying degrees of success, we and others have tried to find associations between indirect markers of infections in early childhood, including antimicrobial use and hospitalisations with infection diagnoses, and subsequent risk of acute lymphoblastic leukemia (15). It has also been shown that acute lymphoblastic leukemia cases had an altered immune response at birth compared to their peers (16). Such research motivated the present study despite the low odds of finding anything conclusive using these indirect noisy data.

### IM and exposures in puberty

This study aimed to find common exposure patterns (*not* e.g., rare genetic variants and acquired immunodeficiency disorders) responsible for the common phenomenon, IM. Very few studies have addressed putative associations between common immunological exposures and EBV-seroprevalence/IM in adolescents and pre-adolescents (11, 28–30). They have found that EBV-seronegative individuals do not appear to have lower levels of circulating antibody to common vaccine antigens and pathogens (28), and that being infected with EBV correlated with being infected with other herpes viruses (notably CMV and HSV-1) (29, 30). Furthermore, socio-demographic proxies for infectious exposures, including sibship constellation, up to and during adolescence are presumably mediated mostly through correlation with the same infectious exposures in the first few years of life, where the highest EBV-seroconversion rates occur (1, 12). Thus, the current contribution fits into a general trend of not finding evidence of associations between common immune system characteristics and IM (9, 11, 28, 31).

Puberty happens very differently in boys and girls and brings with it a maturation of the immune system (3). The key driver of IM occurrence in teen-age years is the IM attack-rate, the probability of primary EBV infection presenting as IM, which is immune-related, increases dramatically at the start of puberty, and varies between the sexes in adolescence but not before (1). We hoped that co-variation in antimicrobial use and IM risk within IM-affected sibships would hint at which parts of the puberty-induced changes in immune competence are relevant to the observed sex differences in the attack rate. We further hoped that this would help explain variation in the attack rate itself, and hence the occurrence of IM. According to this logic, the ideal predictor of IM risk would be an antimicrobial that was used equally much by girls and boys before puberty, but with a frequency of use that would change drastically and differentially between boys and girls at entry into puberty.

*A priori*, it was known that the total use of antimicrobials was similar in boys and girls before puberty, followed by a gentle and steady increase during adolescence for girls compared to boys (22). This we interpret as being due to behavioural differences; girls earlier and to a larger extent manage their own health.

### Strengths and weaknesses

Our results are based on the analysis of readily available infection-related markers of health that seemingly are only weakly associated with both infection proxies (e.g., sibship constellation) (9) and attack rate (as above), and, by implication, with relevant biomarkers for either infections, immune competence or IM risk. This provides a technical explanation as to why the observed effect sizes must be small. Therefore, the analyses of our exposures cannot provide evidence for puberty-induced changes in the immune system as a major component of IM occurrence. Based on our experience with these data and this outcome (1, 9, 12, 22), this is the only noteworthy, but also crucial limitation of this study. E.g., even accommodating somehow the fact that many cases of IM would be unregistered in our IM-affected sibships [30-year risk of IM being 13.3% and 22.4% in males and females, respectively (1), and IM clustering in families (32)], this would not turn our null-finding into a noteworthy signal. There exists no study on the validity of the diagnosis of IM, but it is the only variable used in this study that is likely to be misclassified to a noteworthy degree (17, 19–21, 33). Again, hypothetically fixing this problem would not turn our null-finding into a noteworthy signal, especially because the resultant bias is likely to be non-differential.

Our null finding, which we interpret to represent the workings of common immuno-biological mechanisms in childhood and adolescence should generalize well, at least to other WEIRD (Western, Educated, Industrialised, Rich, Democratic) populations (34), and by the very nature of a null result to other populations as well; to have a signal and yet an overall null-finding would require effects in opposite directions in strata of such a population, which is not plausible.

Theoretical considerations would put small sub-populations of the studied teenagers at an altered risk of IM, but these populations were too small to be of any practical use or interest for this study.

The stratum of girls in 2010+ roughly coincided with a marked decline in HPV-vaccine uptake in 2013 and the following years among girls (35). Specifically, a cohort of girls emerged with debilitating symptoms which they attributed to the HPV vaccine (36). This cohort displayed increased use of health care services and increased health-seeking behaviours in the years surrounding HPV vaccination compared to their peers (36–39). This group likely represents the main component of the above-suggested emergent subcohort of persons with high health care utilization and health-seeking behaviour, spuriously linking recent use of antimicrobials with risk of IM in this stratum. Fatigue was one of the common health complaints in this cohort of girls (36), and is also a common and long lasting consequence of IM (40, 41). Furthermore, EBV infection and IM have also been linked to chronic fatigue syndrome (42). Therefore, it can be assumed, that teenage girls suffering extreme fatigue would seek diagnostic work-up for IM symptoms, and thus end up with an increased incidence of registered/hospital-diagnosed IM.

### Biological interpretation

The most obvious biological interpretation of the results is that the immune response to EBV infection is not, to any noteworthy degree, determined by general features of the host's immune system. Rather, it is governed by specific immunologic pathways. This study reinforces the impression from a diverse array of studies that, with the possible exception of IL-10 genotypes, there are no common immune system characteristics affecting the clinical presentation of late primary EBV infection (9, 11, 28, 31). One of the main hypotheses proposed for explaining why some individuals present with IM upon primary EBV infection, while others do not, is the hypothesis of cross-reactivity; the adolescent host may recruit many cross-reactive memory T-cells previously created in response to other viral infection, which may be more easily activated, but be less efficient, in controlling the infection than primary responses from recruited naïve T-cells (1, 43–45). If this hypothesised mechanism explained a substantial fraction of IM, we would have expected to find a signal in the form of increased antimicrobials use, due to more infections, in future IM cases. However, as Balfour et al. (44), we see no suggestion that this hypothesis is true and, as noted earlier (1), we would find it surprising that cross-reactivity should be a major explanation of such a dramatic change in attack rate by the entry into puberty. However, due to a lack of relevant biomarker measurements, this is clearly an understudied hypothesis (44).

## Conclusions

In conclusion, counts of infection-related health events, such as an antimicrobial prescription, in either a pre-teen or recent three-year exposure window did not predict meaningful variation in risk of IM. This supports the impression that no common immune system characteristics (except perhaps IL-10 genotypes) affect the risk of IM. The hypothesis that cross-reactive memory T cells substantially affect risk of presenting with infectious mononucleosis upon primary EBV infection, must subsequently be considered a little less likely.

## Data Availability

The data analyzed in this study is subject to the following licenses/restrictions: the data used in this study can be accessed through Statistics Denmark (http://www.dst.dk/en/Tilsalg/Forskningsservice) or the Danish Health Data Authority (http://www.sundhedsdatatyrelsen.dk/da/english/health_data_and_registers/research_services), after having obtained the necessary ethical approvals. Requests to access these datasets should be directed to Statistics Denmark, http://www.dst.dk/en/Tilsalg/Forskningsservice.

## References

[1] Rostgaard K Balfour HH Jarrett RF Erikstrup C Pedersen O Ullum H Primary Epstein–Barr Virus infection with and without infectious mononucleosis. PLoS One. (2019) 14:e0226436. 10.1371/journal.pone.0226436 31846480 PMC6917282

[2] Taga K Taga H Tosato G. Diagnosis of atypical cases of infectious mononucleosis. Clin Infect Dis. (2001) 33:83–8. 10.1086/320889 11389499

[3] Ucciferri CC Dunn SE. Effect of puberty on the immune system: relevance to multiple sclerosis. Front Pediatr. (2022) 10:1059083. 10.3389/fped.2022.1059083 36533239 PMC9755749

[4] Koch-Henriksen N Sørensen PS. The changing demographic pattern of multiple sclerosis epidemiology. Lancet Neurol. (2010) 9:520–32. 10.1016/S1474-4422(10)70064-8 20398859

[5] Hjalgrim LL Rostgaard K Engholm G Pukkala E Johannesen TB Ólafsdóttir E Aetiologic heterogeneity in pediatric Hodgkin lymphoma? Evidence from the nordic countries, 1978–2010. Acta Oncol (Madr). (2016) 55:85–90. 10.3109/0284186X.2015.1049660 26073450

[6] Forss A Clements M Bergman D Roelstraete B Kaplan GG Myrelid P A nationwide cohort study of the incidence of inflammatory bowel disease in Sweden from 1990 to 2014. Aliment Pharmacol Ther. (2022) 55:691–9. 10.1111/apt.16735 34907544

[7] Ebert AC Harper S Vestergaard M V Mitchell W Jess T Elmahdi R. Risk of inflammatory bowel disease following hospitalisation with infectious mononucleosis: nationwide cohort study from Denmark. Nat Commun. (2024) 15:8383. 10.1038/s41467-024-52195-8 39333475 PMC11437054

[8] Oreskes N. Why Trust Science? Princeton: Princeton University Press (2019).

[9] Rostgaard K Søegaard SH Stensballe LG Hjalgrim H. Antimicrobials use and infection hospital contacts as proxies of infection exposure at ages 0–2 years and risk of infectious mononucleosis. Sci Rep. (2023) 13:21251. 10.1038/s41598-023-48509-3 38040892 PMC10692188

[10] Jons D Sundström P Andersen O. Targeting Epstein–Barr Virus infection as an intervention against multiple sclerosis. Acta Neurol Scand. (2015) 131:69–79. 10.1111/ane.12294 25208981

[11] Winter JR Jackson C Lewis JE Taylor GS Thomas OG Stagg HR. Predictors of Epstein–Barr Virus serostatus and implications for vaccine policy: a systematic review of the literature. J Glob Health. (2020) 10:010404. 10.7189/jogh.10.010404 32257152 PMC7125428

[12] Rostgaard K Nielsen NM Melbye M Frisch M Hjalgrim H. Siblings reduce multiple sclerosis risk by preventing delayed primary Epstein–Barr Virus infection. Brain. (2023) 146:1993–2002. 10.1093/brain/awac401 36317463

[13] Hjalgrim H Søegaard SH Hjalgrim LL Rostgaard K. Childhood use of antimicrobials and risk of Hodgkin lymphoma: a Danish register-based cohort study. Blood Adv. (2019) 3:1489–92. 10.1182/bloodadvances.2018029355 31072834 PMC6517664

[14] Andersen MA Rostgaard K Niemann CU Hjalgrim H. Antimicrobial use before chronic lymphocytic leukemia: a retrospective cohort study. Leukemia. (2021) 35:747–51. 10.1038/s41375-020-0980-0 32684631

[15] Gradel KO Kaerlev L. Antibiotic use from conception to diagnosis of child leukaemia as compared to the background population: a nested case-control study. Pediatr Blood Cancer. (2015) 62:1155–61. 10.1002/pbc.25477 25790083

[16] Søegaard SH Rostgaard K Skogstrand K Wiemels JL Schmiegelow K Hjalgrim H. Neonatal inflammatory markers are associated with childhood B-cell precursor acute lymphoblastic leukemia. Cancer Res. (2018) 78:5458–64. 10.1158/0008-5472.CAN-18-0831 30217873 PMC7605595

[17] Schmidt M Pedersen L Sørensen HT. The Danish civil registration system as a tool in epidemiology. Eur J Epidemiol. (2014) 29:541–9. 10.1007/s10654-014-9930-3 24965263

[18] Schmidt M Schmidt SAJ Adelborg K Sundbøll J Laugesen K Ehrenstein V The Danish health care system and epidemiological research: from health care contacts to database records. Clin Epidemiol. (2019) 11:563–91. 10.2147/CLEP.S179083 31372058 PMC6634267

[19] Schmidt M Schmidt SAJ Sandegaard JL Ehrenstein V Pedersen L Sørensen HT. The Danish national patient registry: a review of content, data quality, and research potential. Clin Epidemiol. (2015) 7:449–90. 10.2147/CLEP.S91125 26604824 PMC4655913

[20] Wallach Kildemoes H Toft Sørensen H Hallas J. The Danish national prescription registry. Scand J Public Health. (2011) 39:38–41. 10.1177/1403494810394717 21775349

[21] Pottegård A Schmidt SAJ Wallach-Kildemoes H Sørensen HT Hallas J Schmidt M. Data resource profile: the Danish national prescription registry. Int J Epidemiol. (2017) 46:798–798f. 10.1093/ije/dyw213 27789670 PMC5837522

[22] Søegaard SH Spanggaard M Rostgaard K Kamper-Jørgensen M Stensballe LG Schmiegelow K Childcare attendance and risk of infections in childhood and adolescence. Int J Epidemiol. (2023) 52:466–75. 10.1093/ije/dyac219 36413040

[23] Gail MH Wieand S Piantadosi S. Biased estimates of treatment effect in randomized experiments with nonlinear regressions and omitted covariates. Biometrika. (1984) 71:431–44. 10.1093/biomet/71.3.431

[24] Möller S Bliddal M Rubin KH. Methodical considerations on adjusting for Charlson comorbidity Index in epidemiological studies. Eur J Epidemiol. (2021) 36:1123–8. 10.1007/s10654-021-00802-z 34482514

[25] Rostgaard K. Methods for stratification of person-time and events - a prerequisite for poisson regression and SIR estimation. Epidemiol Perspect Innov. (2008) 5:16. 10.1186/1742-5573-5-7 PMC2615420 19014582

[26] Rostgaard K. Simple nested Bayesian hypothesis testing for meta-analysis, cox, poisson and logistic regression models. Sci Rep. (2023) 13:4731. 10.1038/s41598-023-31838-8 36959371 PMC10036629

[27] Husby A Laksafoss AD Thiesson EM Jakobsen KD Andersson M Rostgaard K. EpiForsk: Code Sharing at the Department of Epidemiological Research at Statens Serum Institut. 10.32614/CRAN.package.EpiForsk

[28] Kuri A Jacobs BM Vickaryous N Pakpoor J Middeldorp J Giovannoni G Epidemiology of Epstein–Barr Virus infection and infectious mononucleosis in the United Kingdom. BMC Public Health. (2020) 20:912. 10.1186/s12889-020-09049-x 32532296 PMC7291753

[29] Delaney AS Thomas W Balfour HH. Coprevalence of Epstein–Barr Virus, cytomegalovirus, and herpes simplex virus type-1 antibodies among United States children and factors associated with their acquisition. J Pediatric Infect Dis Soc. (2015) 4:323–9. 10.1093/jpids/piu076 26582871

[30] Sundqvist E Bergström T Daialhosein H Nyström M Sundström P Hillert J Cytomegalovirus seropositivity is negatively associated with multiple sclerosis. Mult Scler. (2014) 20:165–73. 10.1177/1352458513494489 23999606

[31] Kenney AD Dowdle JA Bozzacco L McMichael TM St. Gelais C Panfil AR Human genetic determinants of viral diseases. Annu Rev Genet. (2017) 51:241–63. 10.1146/annurev-genet-120116-023425 28853921 PMC6038703

[32] Rostgaard K Wohlfahrt J Hjalgrim H. A genetic basis for infectious mononucleosis: evidence from a family study of hospitalized cases in Denmark. Clin Infect Dis. (2014) 58:1684–9. 10.1093/cid/ciu204 24696238

[33] Mosbech J Jørgensen J Madsen M Rostgaard K Thornberg K Poulsen TD. The national patient registry. Evaluation of data quality. Ugeskr Laeger. (1995) 157:3741–5. 7631448

[34] Henrich J Heine SJ Norenzayan A. The weirdest people in the world? Behav Brain Sci. (2010) 33:61–83; discussion 83-135. 10.1017/S0140525X0999152X 20550733

[35] Hansen PR Schmidtblaicher M Brewer NT. Resilience of HPV vaccine uptake in Denmark: decline and recovery. Vaccine. (2020) 38:1842–8. 10.1016/j.vaccine.2019.12.019 31918860

[36] Gazibara T Thygesen LC Algren MH Tolstrup JS. Human papillomavirus vaccination and physical and mental health complaints among female students in secondary education institutions in Denmark. J Gen Intern Med. (2020) 35:2647–54. 10.1007/s11606-020-05845-8 32342482 PMC7458962

[37] Mølbak K Hansen ND Valentiner-Branth P. Pre-Vaccination care-seeking in females reporting severe adverse reactions to HPV vaccine. A registry based case-control study. PLoS One. (2016) 11:e0162520. 10.1371/journal.pone.0162520 27611088 PMC5017678

[38] Krogsgaard LW Bech BH Plana-Ripoll O Thomsen RW Rytter D. Hospital contacts and diagnoses five years prior to HPV vaccination among females referred for suspected adverse vaccine effects: a Danish nationwide case-control study. Vaccine. (2019) 37:1763–8. 10.1016/j.vaccine.2019.02.029 30803841

[39] Krogsgaard LW Vestergaard CH Plana-Ripoll O Lützen TH Vestergaard M Fenger-Grøn M Health care utilization in general practice after HPV vaccination-a Danish nationwide register-based cohort study. PLoS One. (2017) 12:e0184658. 10.1371/journal.pone.0184658 28886180 PMC5590983

[40] Odumade OA Hogquist KA Balfour HH. Progress and problems in understanding and managing primary Epstein–Barr Virus infections. Clin Microbiol Rev. (2011) 24:193–209. 10.1128/CMR.00044-10 21233512 PMC3021204

[41] Buchwald DS Rea TD Katon WJ Russo JE Ashley RL. Acute infectious mononucleosis: characteristics of patients who report failure to recover. Am J Med. (2000) 109:531–7. 10.1016/S0002-9343(00)00560-X 11063953

[42] Kristiansen MS Stabursvik J O’Leary EC Pedersen M Asprusten TT Leegaard T Clinical symptoms and markers of disease mechanisms in adolescent chronic fatigue following Epstein–Barr Virus infection: an exploratory cross-sectional study. Brain Behav Immun. (2019) 80:551–63. 10.1016/j.bbi.2019.04.040 31039432

[43] Clute SC Watkin LB Cornberg M Naumov YN Sullivan JL Luzuriaga K Cross-reactive influenza virus-specific CD8+ T cells contribute to lymphoproliferation in Epstein–Barr Virus-associated infectious mononucleosis. J Clin Invest. (2005) 115:3602–12. 10.1172/JCI25078 16308574 PMC1288832

[44] Balfour HH Odumade O Schmeling DO Mullan BD Ed J Knight J Behavioral, virologic, and immunologic factors associated with acquisition and severity of primary Epstein–Barr Virus infection in university students. J Infect Dis. (2013) 207:80–8. 10.1093/infdis/jis646 23100562 PMC3523797

[45] Aslan N Watkin LB Gil A Mishra R Clark FG Welsh RM Severity of acute infectious mononucleosis correlates with cross-reactive influenza CD8T-cell receptor repertoires. MBio. (2017) 8:e01841–17. 10.1128/mBio.01841-17 29208744 PMC5717389

